# Correspondence: Challenging a proposed role for TRPC5 in aortic baroreceptor pressure-sensing

**DOI:** 10.1038/s41467-017-02703-w

**Published:** 2018-03-23

**Authors:** Pratish Thakore, Susan D. Brain, David J. Beech

**Affiliations:** 1 Section of Vascular Biology and Inflammation, School of Cardiovascular Medicine and Research, BHF Cardiovascular Centre of Excellence, King’s College London, London, SE1 9NH UK; 2 Institute of Pharmaceutical Sciences, King’s College London, London, SE1 9NH UK; 3 Leeds Institute of Cardiovascular and Metabolic Medicine, School of Medicine, University of Leeds, Leeds, LS2 9JT UK

## Introduction

Lau et al.^1^ have suggested a role for TRPC5 in the control of blood pressure where they suggest TRPC5 has a pivotal role in baroreflex regulation.

**Fig. 1:**
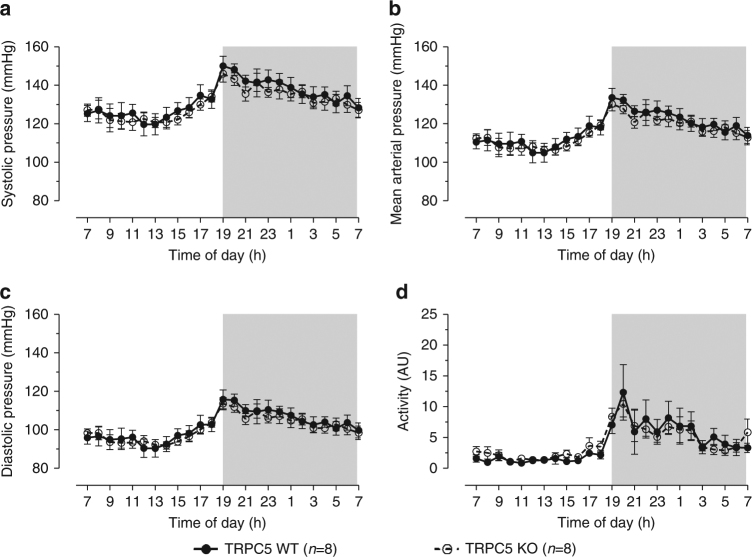
Blood pressure profiling of WT and TRPC5 KO mice. Conscious measurements of **a** systolic, **b** mean arterial and **c** diastolic pressures, and **d** activity were taken over the course of 24 h in WT (wildtype) and TRPC5 KO (knockout) mice implanted with a radio-telemetry probe. Shaded regions depict dark-phase recordings. Data shown as hourly averages and expressed as mean ± s.e.m., and analysed by repeated measures two-way ANOVA followed by the Bonferroni post-hoc correction

As part of this work it was specified that TRPC5 knockout (KO) mice gifted from Professor David Clapham’s laboratory have elevated mean arterial pressure; WT (wildtype) 113 ± 7 mmHg vs. KO 158 ± 26 mmHg (*n* = 12, *p* < 0.05, Fig. 8 in the original paper^1^). In addition, the representative traces from one WT and one KO mouse showed no change in diurnal variation in blood pressure or heart rate, which is unusual (Fig. 8 in ref. ^1^). Furthermore, there was no indication of what time periods correlated to the light and dark phases. The increase in pressure was found not to be associated with an increase in activity, where mouse locomotion was determined by the open field test. Considering that the radio-telemetry probes provided by Data Science International (DSI) can simultaneously record activity along with blood pressure parameters, we found this confusing. This method is widely adopted to show activity-dependent blood pressure correlations. This analysis would have been important as Lau et al.^1^ indicate an increased variability in the BP response of TRPC5 KO mice, indicative of baroreceptor abnormalities. We too are investigating the role of TRPC5 within the cardiovascular system. Our TRPC5 research also involves use of TRPC5 KO mice, kindly gifted by Professor David Clapham. As part of our investigation, we performed independent radio-telemetry experiments to record conscious blood pressure. These experiments were performed using the PA-C10 DSI radio-telemetry system, where catheters were inserted into the left carotid artery before being advanced towards the aortic arch; mice were allowed to recover for a minimum of 2 weeks before conscious measurements of blood pressure and activity over a 24 h period. Our results demonstrate a normal diurnal rhythm in both WT and TRPC5 KO mice (Fig. 1). Furthermore, we found that TRPC5 KO mice have a comparable absolute blood pressure and blood pressure variability to WT counterparts, alongside similar activity levels.

We have corresponded with Professor Xiaoqiang Yao of Lau et al.^1^ to try to resolve these discrepancies. Professor Yao has informed us, after re-calculation of his data, that there was an analytical error. Correcting for this mistake, their results now also show no change in baseline blood pressure between WT and TRPC5 KO mice.

Although disturbance of a mechanical force-sensing mechanism in baroreceptors might not necessarily affect blood pressure, Lau et al.^1^ reported an effect that they defined as severe and suggested that it was explained by a TRPC5 mechanical force-sensing mechanism in the baroreceptors. On the basis of the understanding that they have now changed their position on this, we have asked whether TRPC5 is indeed an important mechanical force sensor of the aortic baroreceptors.

The proposal by Lau et al.^1^ that baroreceptor TRPC5 channels are activated by physiological mechanical force is based primarily on single channel recordings all performed in the presence of lanthanum ions. We are concerned about the inclusion of lanthanum ions because they are non-physiological enhancers of TRPC5 channel activity. On the basis of the data shown, there is no way of knowing whether these TRPC5 channels are mechanically activated in physiological conditions.

Two more approaches were used to test the mechanical force dependence in which whole-cell currents were measured without lanthanum ions; in one case the extracellular medium was made hypo-tonic, in the other positive pressure was applied to the cell interior. In neither case is it shown that the procedure caused a mechanical change or that there was relevance to mechanical force experienced by baroreceptors in vivo. This aside, the induced currents did not have the distinctive current–voltage relationship (IV) of TRPC5 channels. It is stated that the IV had the double-rectifying shape expected for TRPC5 channels, but this shape is not evident in the data (Fig. 6f in ref. ^1^). Moreover, the inward current appears not to have been significantly affected by TRPC5 KO (Fig. 6h in ref. ^1^); it is unclear why this would be the case when the outward current was affected and the T5E3 antibody was inhibitory.

The authors refer to the baroreceptor channels both as TRPC5 channels (homomers) and TRPC5-containing channels (heteromers). This matter is not consistently handled but it seems that the authors’ preference is for heteromers even though they exclude established partners of TRPC5. We surmise that the reason for this preference is that the IV was not as expected for TRPC5 channels in the absence of lanthanum ions (Figs. 2a and 6f in ref. ^1^). However, we suggest that the single channel data were almost certainly from TRPC5 channels because the unitary conductance in the presence of 20 μM lanthanum ions was 25 pS. Not specified in the paper is that TRPC5 channels have conductance of 40 pS in the absence of lanthanum ions and about 25 pS in 20 μM lanthanum ions^2,3^. It is therefore unlikely that the baroreceptor single channel data are explained by anything other than TRPC5 channels, which were induced by lanthanum ions (i.e. homomers). It could be that in the absence of lanthanum ions the current was mediated mostly by something other than TRPC5 channels; channels which had a relatively linear IV. Some of this current may have been due to TRPC5 heteromers but the amplitude of this current or its mechanical sensitivity is presumably insufficient to significantly affect blood pressure (because TRPC5 KO does not affect blood pressure).

Overall, we recognise that Lau et al.^1^ have performed a large number of experiments, many of which would have been challenging, and that multiple efforts were made to show relevance of TRPC5. However, we are concerned that the authors’ reanalysis of their data has led them to reach a different conclusion from that presented in the paper. In light of this it would seem advisable to re-analyse data sets to be sure the findings are accurately presented. Moreover, we are concerned that experimental protocols and presentation style have favoured conclusions about the importance of TRPC5 channels in this biology.

We suggest that further studies are needed before it can be concluded that TRPC5 is important in cardiovascular regulation or that TRPC5 channels are activated by physiological mechanical force in baroreceptors—at least importantly relative to other mechanisms. Lau et al.^1^ discuss that results from a prior study conducted under more physiological conditions are not consistent with their conclusions^4^. Indeed it will be interesting to investigate the channels in pathophysiological conditions. We would welcome further input and comment from the authors and input from others within and outside the immediate field of investigation.

## Methods

### Animals

All experiments were carried out in accordance with the Animal (Scientific Procedures) Act 1986 and approved by the King’s College London Animal Care and Ethics Committee, and complied Animal Research: Reporting of In Vivo Experiments (ARRIVE) guidelines^5^. Animals were housed in a climatically controlled environment (22 ± 2 °C) under a 12 h light (7am–7pm)/dark (7pm–7am) cycle with access to chow and water ad libitum. WT and global TRPC5 KO mice were kindly gifted by Professor David E. Clapham (Harvard Medical School, USA)^6^. TRPC5 KO mice were bred in-house where age- and sex-matched littermates (8–12 weeks, 20–30 g) were used for investigation purposes.

### Radio-telemetry of conscious blood pressure

Baseline haemodynamics were assessed in animals using PA-C10 radio-telemetry devices (Data Science International, USA)^7^. Briefly, mice were anaesthetised with isoflurane 1.5–2% isoflurane (Isocare, Abbott Laboratories, UK) carried in 95% O_2_/5% CO_2_ (The BOC Group, UK) and preoperative analgesia was provided (50 µg kg^−1^ buprenorphine, i.m., Vetergesic, Alstoe Animal Health, UK). Under aseptic conditions, the catheter of the telemetry device was surgically inserted into the left common carotid artery and advanced towards the aorta before being secured with 5-0 non-absorbable silk sutures threads (Mersilk, Ethicon, USA). The body of the telemetry device was placed in a s.c. pocket in the right flank. Mice were allowed to recover for 2 weeks before recording the baseline blood pressures and activity over a 24-h period.

### Statistical analysis

Investigators were blinded to the study groups where appropriate. All data were analysed in GraphPad Prism software (version 5.04, GraphPad Software Inc., USA). All values are illustrated as mean ± standard error of the mean (s.e.m.) and *n* represents the number of animals. Time course data were analysed using repeated measures two-way ANOVA followed by the Bonferroni post-hoc correction. *p* < 0.05 was regarded as statistically significant.

### Data availability

The data sets generated during and/or analysed for the current study are available from the corresponding author on reasonable request.
